# The Indian Ocean Dipole and Cholera Incidence in Bangladesh: A Time-Series Analysis

**DOI:** 10.1289/ehp.1002302

**Published:** 2010-10-27

**Authors:** Masahiro Hashizume, A.S.G. Faruque, Toru Terao, Md Yunus, Kim Streatfield, Taro Yamamoto, Kazuhiko Moji

**Affiliations:** 1 Institute of Tropical Medicine and the Global Center of Excellence Program, Nagasaki University, Nagasaki, Japan; 2 International Centre for Diarrhoeal Disease Research, Bangladesh, Dhaka, Bangladesh; 3 Faculty of Education, Kagawa University, Kagawa, Japan; 4 Research Institute for Humanity and Nature, Kyoto, Japan

**Keywords:** Bangladesh, cholera, El Niño-Southern Oscillation, Indian Ocean dipole, time-series analysis

## Abstract

**Background:**

It has been reported that the El Niño–Southern Oscillation (ENSO) influences the interannual variation of endemic cholera in Bangladesh. There is increased interest in the influence of the Indian Ocean dipole (IOD), a climate mode of coupled ocean–atmosphere variability, on regional ocean climate in the Bay of Bengal and on Indian monsoon rainfall.

**Objectives:**

We explored the relationship between the IOD and the number of cholera patients in Bangladesh, controlling for the effects of ENSO.

**Methods:**

Time-series regression was performed. Negative binomial models were used to estimate associations between the monthly number of hospital visits for cholera in Dhaka and Matlab (1993–2007) and the dipole mode index (DMI) controlling for ENSO index [NINO3, a measure of the average sea surface temperature (SST) in the Niño 3 region], seasonal, and interannual variations. Associations between cholera cases and SST and sea surface height (SSH) of the northern Bay of Bengal were also examined.

**Results:**

A 0.1-unit increase in average DMI during the current month through 3 months before was associated with an increase in cholera incidence of 2.6% [(95% confidence interval (CI), 0.0–5.2; *p* = 0.05] in Dhaka and 6.9% (95% CI, 3.2–10.8; *p* < 0.01) in Matlab. Cholera incidence in Dhaka increased by 2.4% (95% CI, 0.0–5.0; *p* = 0.06) after a 0.1-unit decrease in DMI 4–7 months before. Hospital visits for cholera in both areas were positively associated with SST 0–3 months before, after adjusting for SSH (*p* < 0.01).

**Conclusions:**

These findings suggest that both negative and positive dipole events are associated with an increased incidence of cholera in Bangladesh with varying time lags.

Cholera remains a major public health problem in many places, including Bangladesh and India, as well as a number of countries in Africa and South America (Sack et al. 2006). *Vibrio cholerae*, the bacterium that causes the disease, is known to inhabit riverine, estuarine, and coastal ecosystems consisting of abiotic factors (e.g., temperature, sunlight, pH, salinity), phytoplankton, aquatic plants, and copepod zooplankton (Colwell et al. 1977; Huq et al. 1983; Islam et al. 1994). *V. cholerae* has an increased growth rate in aquatic environments with warmer temperatures, particularly in combination with a high pH and blooms of phytoplankton, aquatic plants, or algae (Cockburn and Cassanos 1960; Colwell 1996; Islam et al. 1989). The growth of phytoplankton and aquatic plants is influenced by sunlight, temperature, and nutrient availability. This in turn alters the dissolved oxygen and carbon dioxide content of the water, and thus the pH of the water. High levels of phytoplankton provide a food source for zooplankton to which *V. cholerae* attach, thus protecting the bacteria from the external environment and allowing their proliferation (Lipp et al. 2002). Ingestion of a few copepods that carry a high concentration of *V. cholerae* can initiate an infection (Lipp et al. 2002), and this occurs more frequently when untreated water is consumed.

It has been reported that the El Niño– Southern Oscillation (ENSO) plays a role in the interannual variation of endemic cholera in Bangladesh (Bouma and Pascual 2001; Colwell 1996; Pascual et al. 2000; Rodó et al. 2002). Sea surface temperature (SST) and sea surface height (SSH) in the Bay of Bengal have been proposed to influence the incidence of cholera in Dhaka (Colwell 1996; Koelle et al. 2005; Lobitz et al. 2000). One study, however, observed no association between SST or SSH and the incidence of cholera in Matlab, in rural Bangladesh (Emch et al. 2008). A recent study in Matlab and Kolkata, India, reported that satellite- derived data of chlorophyll concentration, an indicator of phytoplankton levels, in the Bay of Bengal can successfully predict cholera incidence (Constantin de Magny et al. 2008). The strong correlation between SST in the Bay of Bengal and the outbreak of cholera may occur because the warm waters along the coast, coupled with plankton blooms driven by warm ocean temperatures, are favorable for *V. cholerae* proliferation (Lipp et al. 2002; Lobitz et al. 2000). SST over the entire basin of the Indian Ocean is uniformly modulated by the ENSO after a time lag (Wallace et al. 1998).

The Indian Ocean dipole (IOD) is a climate mode arising from an ocean–atmosphere interaction that causes interannual climate variability in the tropical Indian Ocean (Saji et al. 1999; Webster et al. 1999). A positive IOD indicates SST anomalies with warmer than usual SSTs over the western basin and cooler than usual SSTs in the eastern basin near Sumatra. A negative IOD occurs when the SST is anomalously warm in the eastern basin and anomalously cold in the western tropical Indian Ocean. Although the extent to which the IOD is independent of ENSO has been debated (Jensen 2007), there is growing evidence that this air–sea interaction is specific to the Indian Ocean (Behera et al. 2006; Fischer et al. 2005; Hong et al. 2008).

The IOD has been reported to affect regional ocean climate (Yamagata et al. 2004). Han and Webster (2002) reported that IOD events strongly influence sea level variations in the Bay of Bengal and that sea level anomalies in the northern bay may predict flooding and outbreaks of cholera in Bangladesh. Lobitz et al. (2000) observed that variability in sea level correlates with the number of hospital visits for cholera in Dhaka. They also reported that rising SSH may indicate inland incursion of plankton-laden water from the Bay, which acts as a reservoir of *V. cholerae*. The IOD also plays an important role as a modulator of the Indian monsoon rainfall (Ajayamohan and Rao 2008; Ashok et al. 2001, 2004). Several researchers have also reported that rainfall and associated river levels influence cholera patterns in Bangladesh (Akanda et al. 2009; Hashizume et al. 2008). However, the effect of IOD on cholera incidence has not previously been examined. Clarifying the effect of IOD could assist in the development of accurate early warning systems for cholera epidemics and aid in disease control. Here we report on the relationship between the IOD and the incidence of cholera in Dhaka and Matlab, Bangladesh, using time-series methodology controlling for the effects of ENSO. We used satellite-derived data to quantify the strength and mode of the IOD and ENSO and to determine SST and SSH in the northern Bay of Bengal over a 15-year period, from January 1993 to December 2007.

## Materials and Methods

### Hospital surveillance

The primary outcome of this study was the monthly number of patients with cholera who were treated at the International Centre for Diarrhoeal Disease Research, Bangladesh (ICDDR,B) hospitals in Dhaka and Matlab. The ICDDR,B hospital in Dhaka provides health care services to a large urban population within the city and gives free treatment for > 100,000 cases of diarrhea each year. From 1993 to 1995, every 25th patient who visited the hospital was enrolled in a surveillance system, and from 1996 to 2007, every 50th patient was enrolled. Matlab is a riverine area situated approximately 50 km southeast of Dhaka. The ICDDR,B has maintained a health and demographic surveillance system (HDSS) in Matlab since 1966 and has registered births, deaths, and migrations for the area from that time. In 2002, 142 villages with a population > 220,000 people were included in the HDSS (ICDDR,B 2006). Every hospital visit of patients who resided in the HDSS has been registered in the hospital surveillance system.

### Case ascertainment

A stool sample was routinely collected from patients enrolled in the surveillance system and was microbiologically examined to identify common enteric pathogens, including *V. cholerae*. Information regarding the date of the hospital visit and the pathogens identified from each stool specimen during a 15-year period (from January 1993 to December 2007) was retrieved from the database of the hospital surveillance system. Monthly total cholera cases were calculated and analyzed.

### Ocean climate data

The strength of the IOD was measured using the dipole mode index (DMI), defined as the difference in SST between the western (10°S–10°N, 50–70°E) and eastern (10°S–0°, 90–110°E) tropical Indian Ocean (Saji et al. 1999). We calculated DMI values using SST data derived from the U.S. National Oceanic and Atmospheric Administration (NOAA) Optimum Interpolation Sea Surface Temperature data set (Reynolds and Smith 1994; Reynolds et al. 2002). The strength of ENSO was represented by the NINO3 index, a measure of the average SST in the Niño 3 region (5°S–5°N, 90–150°W) of the Pacific Ocean, which was derived from NOAA Climate Prediction Center data sets (NOAA Climate Prediction Center 2009).

Mean monthly SSTs in the Bay of Bengal (20–21°N, 90–91°E) were derived from the NOAA Optimum Interpolation Sea Surface Temperature data set (Reynolds and Smith 1994; Reynolds et al. 2002). From 1993 through 2002, the TOPEX/Poseidon sensor was used to measure mean monthly SSHs in the same area [National Aeronautics and Space Administration (NASA) Jet Propulsion Laboratory 2009b] and from 2003 through 2007, the Jason-1 sensor was used (aNASA Jet Propulsion Laboratory 2009a).

### Statistical analysis

We examined the relationship between the number of cholera cases per month and DMI and NINO3 using negative binomial generalized linear models (GLMs) (McCullagh and Nelder 1989). The primary analysis examined whether a change in the DMI for a given month was associated with a change in the number of cholera cases *n* months later. Temporal associations between climate and disease can be confounded by temporal trends and seasonal patterns. To account for seasonality in the incidence of cholera that is not directly linked with the IOD, indicator variables for months were included in the model. Indicator variables for the years of the study were also incorporated into the model to allow for long-term trends and other variation between the years. To allow for autocorrelations, an autoregressive term at order 1 was incorporated into the models (Brumback et al. 2000).

### Models for DMI and NINO3

Based on exploratory analyses and data from the literature, we considered lag times (the delay in the effect of DMI and NINO3 on the number of cholera cases) of up to 11 months. In our initial analyses, a natural cubic spline (3 df) (Durrleman and Simon 1989) was fitted to the average DMI and NINO3 during three 4-month periods (i.e. lags 0–3, 4–7, and 8–11 months) as separate splines that were simultaneously included in the model. Natural cubic splines were used to create graphs of the predicted numbers of cholera cases plotted as smoothed functions of DMI and NINO3 (Durrleman and Simon 1989). These graphs were used to visually assess whether the functional form of the adjusted relationship appeared to be linear across the full range of independent variables. The details of the final model are described in the Supplemental Material (doi:10.1289/ehp.1002302). Because spring and fall cholera peaks have potentially different explanatory drivers, the analysis was repeated restricting data separately to January–June and July–December.

If the overall association with DMI or NINO3 was significant, we fitted the data using both linear models and linear threshold models (Armstrong 2006) that assumed a log-linear increase in risk above a threshold and no increase in risk below the threshold (or vice versa), with thresholds selected based on maximum likelihood estimations over a grid of all possible one decimal point values for DMI or NINO3 within a range indicated on the exposure–cholera graphs. When the deviance estimates for the linear and the best-fitted threshold model differed by less than 3.84 (χ^2^ for 1 df at the *p* = 0.05 level), we chose the simpler linear model. The increase in the number of cholera cases associated with a 0.1-unit increase in DMI or NINO3 (estimated as coefficients from the regression model) was reported as a percentage change with corresponding 95% confidence intervals (CIs).

### Models for SST and SSH

A secondary analysis was conducted to examine the relationship between the number of cholera cases and SST and SSH in the Bay of Bengal. This analysis was necessary to gain insight into the possible causal pathways between the IOD and the incidence of cholera. A natural cubic spline (3 df) was fitted to the average SST and SSH over lag times of 0–3 months. The final model is described in the Supplemental Material (doi:10.1289/ehp.1002302).

Finally, we estimated the relationship between DMI and the number of cholera cases using models adjusted for NINO3, SST, and SSH to clarify which component of the DMI–cholera association was operating through factors associated with SST and SSH. To investigate whether the results were sensitive to the levels of control for seasonal patterns, analyses were repeated using Fourier terms of the month up to the fifth harmonics per year, adding one harmonic at a time. Statistical significance was set at *p* < 0.05.

## Results

**Figure 1:**
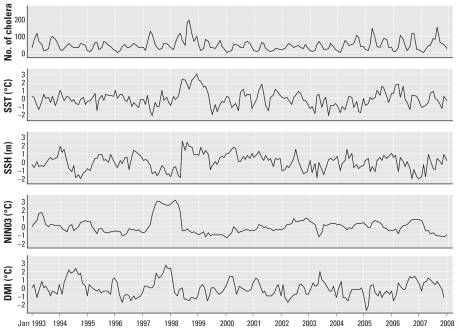
Time series for the number of cholera patients each month in ICDDR,B Hospital in Dhaka, standardized anomalies of the SST and SSH in the Bay of Bengal, the NINO3 index (the SST in the NINO3 region), and the DMI relative to the 1993–2007 mean for each variable. Standardized anomalies were calculated only for the descriptive analysis; raw data were used for the regression analysis.

Figure 1 shows the time series for the number of cholera patients per month in Dhaka, the SST and SSH anomalies in the northern Bay of Bengal, and the DMI and NINO3 during the study period [see also Supplemental Material, Figure 1, for Matlab (doi:10.1289/ehp.1002302)]. Strong positive IOD events occurred in 1994 and 1997, during which time the DMI peaked in August and October, respectively. We observed strong ENSO events (indicated by large NINO3 index values) in 1997–1998. In 1998, we observed exceptionally high SST and SSH, preceding a sharp increase in the number of cholera patients in Dhaka (Figure 1).

**Figure 2:**
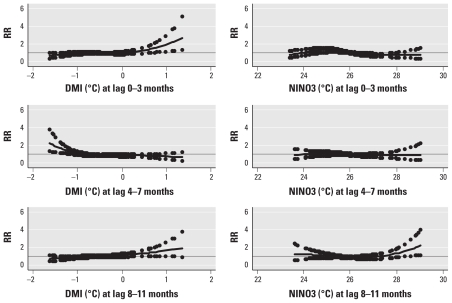
Relationships between the relative risk (RR) of cholera scaled to the mean monthly number of patients in Dhaka hospital and the DMI and the NINO3 index adjusted for potential mutual confounding. The center line in each graph shows the estimated spline curve, and the upper and lower dots represent 95% CIs. The solid horizontal line indicates RR = 1.

The GLM adjusted for potential mutual confounding between DMI and NINO3 revealed that the number of hospital visits for cholera in Dhaka was increased when the mean DMI during the current month and 3 months before (lag 0–3 months) was positive and when the mean DMI during the fourth through the seventh month before (lag 4–7 months) was negative. The number of monthly hospital visits in Dhaka was also increased in association with a positive mean NINO3 index over the eighth through the eleventh month before (lag 8–11 months; Figure 2). Linear model estimates indicated that the number of cholera patients in Dhaka increased by 2.6% (95% CI, 0.0–5.2; *p* = 0.05) with a 0.1-unit increase in average DMI during the previous 0–3 months, and increased by 2.4% (95% CI, 0.0–5.0; *p* = 0.06) with a 0.1-unit decrease in average DMI 4–7 months before (Table 1). In addition, cholera cases increased by 1.4% (95% CI, −0.5 to 3.2; *p* = 0.15) with a 0.1-unit increase in the mean NINO3 index 8–11 months before.

We observed associations with positive DMI 0–3 months before, negative DMI 4–7 months before, and NINO3 8–11 months before mainly for cholera cases that occurred in January–June (data not shown). In contrast with findings for both seasons combined, cases diagnosed in July–December were increased in association with positive DMI 8–11 months before.

Risk–response patterns in Matlab were similar to those in Dhaka [see also Supplemental Material, Figure 2 (doi:10.1289/ehp.1002302)], except that we did not observe the inverse association with low DMI 4–7 months before in Matlab. The linear model suggested that the number of cholera patients in Matlab increased by 6.9% (95% CI, 3.2–10.8; *p* < 0.01) with a 0.1-unit increase in mean DMI during the previous 0–3 months, and by 4.7% (95% CI, 2.2–7.3; *p* < 0.01) with a 0.1-unit increase in NINO3 at a lag of 8–11 months (Table 1). Seasonal analysis indicated that the association with positive DMI during the previous 0–3 months occurred mainly for cases diagnosed in July–December. Average NINO3 during the lag of 8–11 months was not significantly associated with hospital visits in either season (*p* > 0.05).

**Figure 3:**
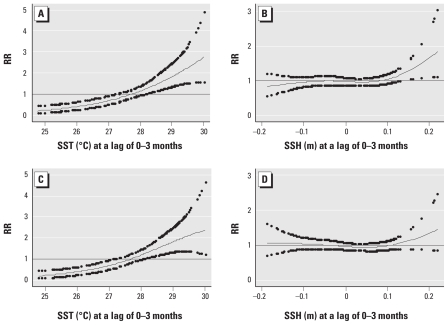
Relationships between the relative risk (RR) of cholera scaled to the mean monthly number of patients seen at the Dhaka hospital and SST and SSH in the Bay of Bengal, unadjusted (*A*,*B*) and adjusted (*C*,*D*) for potential mutual confounding between SST and SSH. The center line in each graph shows the estimated spline curve, and the upper and lower dots represent 95% CIs. The solid horizontal line indicates RR = 1.

The average number of monthly hospital visits for cholera was positively associated with SST and SSH during the previous 0–3 months [Figure 3A,B; see also Supplemental Material, Figure 3A,B, for Matlab (doi:10.1289/ehp.1002302)]. Adjusting for potential mutual confounding between SST and SSH had little effect on the response pattern for SST (Figure 3C) but almost eliminated the association with SSH in both areas [Figure 3D; see Supplemental Material, Figure 3C,D, for Matlab (doi:10.1289/ehp.1002302)]. Each 0.1°C increase in monthly SST during the previous 0–3 months was associated with a 4.8% (95% CI, 2.4–7.3; *p* < 0.01) increase in the number of cholera patients in Dhaka and a 9.0% (95% CI, 5.3–12.9; *p* < 0.01) increase in Matlab.

After adjustment for SST and SSH, positive associations with higher DMI during the previous 0–3 months slightly decreased both in Dhaka (1.9% more cases; 95% CI, −1.8 to 5.6; *p* = 0.33) and in Matlab (4.3% more cases; 95% CI, −0.9 to 9.7; *p* = 0.10). Estimated effects of DMI and NINO3 remained largely unaltered after incorporating Fourier terms of two to five harmonics (added one at a time to the model) in place of indicator variables for months. In general, Akaike’s information criterion and deviance values for models of associations were smaller with DMI than with NINO3 in Dhaka, and were smaller with NINO3 than with DMI in Matlab [see Supplemental Material, Figure 4 and Table 1 (doi:10.1289/ehp.1002302)]. These results suggest that DMI is a better predictor of cholera in Dhaka and that NINO3 is a better predictor in Matlab.

## Discussion

The present analyses indicate that hospital visits for cholera in both Dhaka and Matlab increased in association with a positive IOD (positive DMI) during the previous 0–3 months and with an increase in the ENSO (high NINO3 index) during the previous 8–11 months. In addition, we observed an association between cholera cases and a negative IOD during the previous 4–7 months in Dhaka but not in Matlab.

The ENSO has been reported to play an important role in the interannual variation of endemic cholera in Bangladesh (Bouma and Pascual 2001; Colwell 1996; Pascual et al. 2000; Rodó et al. 2002). There is evidence that interannual variability of cholera incidence between 1980 and 1998 in Dhaka is positively associated with NINO3.4 (average SST in Niño 3.4 region, 5°S–5°N, 120–170°W) (Pascual et al. 2000). An analysis of historical data from Bengal (1891–1940) showed a significant positive correlation between spring cholera mortality in coastal regions and monthly SST in the Bay of Bengal (Bouma and Pascual 2001). Research has shown that the positive association between cholera and ENSO was apparent in 1980–2001 but was weaker in the preceding century (Rodó et al. 2002).

The physical mechanisms underlying the association between ENSO and cholera incidence have not been fully elucidated. Cash et al. (2008) demonstrated that winter ENSO events lead to a general warming of the tropical atmosphere that persists into the next summer. This warming leads to a change in the circulation over the Indian Ocean region, which in turn leads to greater moisture convergence and increased monsoon rainfall over Bangladesh. However, this model did not account for the internal dynamics of the Indian Ocean, which may play an important role in influencing the regional climate in Bangladesh. Cash et al. (2009) also reported that the state of the Indian Ocean during the summer months is closely associated with the regional climate of Bangladesh, independent of ENSO. They concluded that Indian Ocean SST anomalies might therefore represent an independent predictor of the monsoon and cholera epidemics.

The IOD has been suggested to strongly influence sea level variations in the Bay of Bengal, which is linked with the water level in the area affected by tidal waves (Han and Webster 2002). Sea level changes in the northern Bay are driven predominantly by the effect of equatorial wind variability (Han and Webster 2002). Because westerly wind changes at the equator influence the sea level in the eastern equatorial ocean during summer and fall, and a high sea level in the eastern equatorial ocean is generally associated with a negative dipole in the tropical Indian Ocean, a negative dipole event tends to enhance flooding in Bangladesh. In contrast, a positive dipole tends to result in a lower sea level in the eastern equatorial ocean, which is related to a lower sea level in the northern Bay. The lag between the occurrence of positive/negative dipole mode and sea level anomalies in the northern bay is still unclear, but may be short given that the correlation between wind changes at the equator and sea level anomalies is observed with a time lag of < 1 month (Han and Webster 2002).

The IOD also plays an important role as a modulator of the Indian monsoon rainfall. It has been previously reported that when the ENSO–Indian summer monsoon rainfall (ISMR) correlation was strong between the late 1960s and late 1970s, the IOD–ISMR correlation was weak. By the late 1980s the ENSO–ISMR correlation weakened whereas the IOD–ISMR correlation strengthened rapidly (Ashok et al. 2001). Atmospheric general circulation modeling studies have reported that the IOD phenomenon reduces the influence of ENSO on the Indian monsoon by inducing an anomalous divergence center in the eastern tropical Indian Ocean, leading to convergence over the Bay of Bengal and the Indian monsoon region (Ashok et al. 2004). Ajayamohan and Rao (2008) reported a notable shift in the early 1980s and suggested that the extreme rainfall events in the Ganges-Mahanadi Basin in India, upstream of Bangladesh, are modulated by coupled ocean–atmosphere conditions associated with a positive IOD. The present study period follows this shift. The possible link between IOD and cholera through the increased river levels due to high rainfall in the upstream is also worthy of further investigation.

In the present analysis, the estimated effect of the IOD on cholera incidence diminished after we adjusted for SST and SSH in the Bay of Bengal. This finding suggests that the effect of the IOD on cholera incidence may be partially explained by its effect on SST and SSH. Although the causal pathways are thought to be very complicated, high river levels/flooding are likely to be one of the important causal pathways. Increased river levels and flooding adversely affect water sources and sewerage systems and increase the exposure to water contaminated with *V. cholerae*. The possible link between flooding and cholera may also be associated with the growth and multiplication of *V. cholerae*, because flooding increases the level of insoluble iron, which in turn improves the survival rate of *V. cholerae* (Lipp et al. 2002). It has also been suggested that flooding washes away the vibriophages that prey on *V. cholerae*, resulting in increased concentration of the bacterium in the water (Faruque et al. 2005), although a recent simulation study did not support this hypothesis (King et al. 2008). It is unlikely that these possible pathways explain all cholera cases during the monsoon season, because the incidence of cholera often dips during monsoon flooding. This dip is thought to result from a reduction in salinity levels due to increased river discharge, which reduces the survival of *V. cholerae* and decreases its concentration by monsoon rainfall (dilution effect) (Pascual et al. 2002).

We observed the association between negative dipoles and increased cholera incidence in Dhaka but not in Matlab. The reason for this difference is unclear and warrants further investigation. Given that the impact of negative dipoles on cholera incidence may operate through increased SSH and river levels, this finding is not in accord with the findings that the effects of SST and SSH were larger in Matlab than in Dhaka. This is compatible with the fact that Matlab is closer to the Bay of Bengal and is affected by tidal level. A difference in population density between the two areas that results in different transmission dynamics, hygiene and sanitation conditions, and behavioral patterns may explain the different findings between these two areas. Alternatively, it could be due to error or misclassification, or lower statistical power in Matlab.

A limitation of this study is that we did not examine the effect of population immunity, which is dynamic and changes over time along with the population at risk. However, we do not believe that this would have materially altered the results, because infection with *V. cholerae* increases immunity to reinfection that lasts at least more than a year (Koelle et al. 2005). Hence, it is unlikely to have obscured the intraannual association of cholera on the factors investigated in this study.

Although the present findings do not directly represent a causal connection, they do suggest that Indian Ocean SST variability should be taken into account when building predictive models for cholera using ocean-climate data. Because of the serious global consequences of cholera and its sensitivity to climate, the World Health Organization has proposed developing an early warning system for cholera epidemics using climatic parameters (World Health Organization 2005). Although attempts to predict the incidence of cholera using ocean-climate data from preceding months have been made (Akanda et al. 2009; Constantin de Magny et al. 2008), an accurate climate-based prediction of cholera epidemics with a longer lag time has not yet been developed. A system for forecasting IOD has been developed, and IOD events are predictable 4 months in advance (Luo et al. 2007). Combined with such a prediction model, the results of this study provide a basis for predicting cholera epidemics in Bangladesh and have the potential to improve disease control in vulnerable areas. Because geographic variability of the association may be high, these results indicate that the role of IOD on incidence of cholera in other places is also worthy of investigation.

## Figures and Tables

**Table 1:** Estimates for linear association between cholera cases and DMI and NINO3 in Dhaka and Matlab: percent change in the number of cholera cases for 0.1 increase in DMI and NINO3.

	Dhaka						Matlab					
	DMI (˚C)			NINO3 (˚C)			DMI (˚C)			NINO3 (˚C)		
Lag (months)	Percent change	95% CI	*p*-Value	Percent change	95% CI	*p*-Value	Percent change	95% CI	*p*-Value	Percent change	95% CI	*p*-Value
0–3	2.6	0.0 to 5.2	0.05	—	—	—	6.9	3.2 to 10.8	< 0.01	—	—	—
4–7	−2.4	−15.0 to 0.0	0.06	—	—	—	—	—	—	—	—	—
8–11	—	—	—	1.4	−0.5 to 3.2	0.15	—	—	—	4.7	2.2 to 7.3	< 0.01

Models were adjusted for potential mutual confounding between DMI and NINO3, seasonal variation (indicator variables for the month), interannual variation (indicator variables for the year), and first-order autoregressive term. —, “not quantified“ because overall association was not significant (*p* > 0.05).
